# Confronting challenges to opioid risk mitigation in the U.S. health system: Recommendations from a panel of national experts

**DOI:** 10.1371/journal.pone.0234425

**Published:** 2020-06-15

**Authors:** Erin P. Finley, Suyen Schneegans, Megan E. Curtis, Vikhyat S. Bebarta, Joseph K. Maddry, Lauren Penney, Don McGeary, Jennifer Sharpe Potter

**Affiliations:** 1 UT Health San Antonio, San Antonio, Texas, United States of America; 2 South Texas Veterans Health Care System, San Antonio, Texas, United States of America; 3 University of Colorado School of Medicine, Aurora, CO, United States of America; 4 Emergency Department, Brooke Army Medical Center, San Antonio, Texas, United States of America; 5 59th Medical Wing Science and Technology Cell, San Antonio, Texas, United States of America; 6 San Antonio Uniformed Services Health Education Consortium, San Antonio, Texas, United States of America; US Army Engineer Research and Development Center, UNITED STATES

## Abstract

**Background:**

Amid the ongoing U.S. opioid crisis, achieving safe and effective chronic pain management while reducing opioid-related morbidity and mortality is likely to require multi-level efforts across health systems, including the Military Health System (MHS), Department of Veterans Affairs (VA), and civilian sectors.

**Objective:**

We conducted a series of qualitative panel discussions with national experts to identify core challenges and elicit recommendations toward improving the safety of opioid prescribing in the U.S.

**Design:**

We invited national experts to participate in qualitative panel discussions regarding challenges in opioid risk mitigation and how best to support providers in delivery of safe and effective opioid prescribing across MHS, VA, and civilian health systems.

**Participants:**

Eighteen experts representing primary care, emergency medicine, psychology, pharmacy, and public health/policy participated.

**Approach:**

Six qualitative panel discussions were conducted via teleconference with experts. Transcripts were coded using team-based qualitative content analysis to identify key challenges and recommendations in opioid risk mitigation.

**Key results:**

Panelists provided insight into challenges across multiple levels of the U.S. health system, including the technical complexity of treating chronic pain, the fraught national climate around opioids, the need to integrate surveillance data across a fragmented U.S. health system, a lack of access to non-pharmacological options for chronic pain care, and difficulties in provider and patient communication. Participating experts identified recommendations for multi-level change efforts spanning policy, research, education, and the organization of healthcare delivery.

**Conclusions:**

Reducing opioid risk while ensuring safe and effective pain management, according to participating experts, is likely to require multi-level efforts spanning military, veteran, and civilian health systems. Efforts to implement risk mitigation strategies at the patient level should be accompanied by efforts to increase education for patients and providers, increase access to non-pharmacological pain care, and support use of existing clinical decision support, including state-level prescription drug monitoring programs.

## Introduction

Over the past twenty-five years, opioid analgesics have been commonly prescribed in the U.S. for acute and chronic pain management [1]. In response to growing evidence that use of opioids for management of chronic pain may not be effective and is associated with significant risk of developing opioid use disorder, death by overdose, and other health conditions [1–4], U.S. healthcare and governmental organizations have taken a variety of steps to reduce opioid prescribing and overdose risk. Examples include implementing new policies for opioid prescribing in Massachusetts [5], disseminating clinical practice guidelines (e.g., CDC Guidelines for Prescribing Opioids for Chronic Pain) [6,7], and using prescription drug monitoring programs (PDMPs) [8] and other clinical decision support (CDS) to aid in risk assessment and management [9,10].

Although overall rates of opioid prescribing have declined since 2011 [11], opioid-related overdose deaths continue to increase suggesting that simply reducing opioid use in clinical care may not reduce opioid-related mortality [12]. In 2017 alone, 47,600 persons died in the U.S. from overdoses involving opioids, accounting for 67.8% of all drug overdose deaths [12]. For this and other reasons, the debate continues over whether there is an appropriate role for opioid medications within chronic pain management [13]. Efforts to reduce opioid-related harms face the additional challenge of healthcare fragmentation, with the Military Health System (MHS), Department of Veterans Affairs (VA), and public and private civilian health systems operating within unique organizational structures independently treating specialized but frequently overlapping patient populations. For example, the patient populations for military, VA, and civilian healthcare systems are in many ways distinct. Service members are on average considerably younger than Veterans, a higher preponderance of men receive care in military and VA settings, and individuals relying on VA health care tend to have more health conditions than those who do not [14–16]. At the same time, there is also frequent overlap and intersection between the patient populations served by these systems, particularly when considering that many Veterans receive some or most of their care in military or civilian treatment facilities, and many of those receiving care in military treatment facilities are non-Veteran dependents or retirees who may also be receiving care from outside the military system. Relatively little of the health and policy literature has been devoted to considering the unique and shared challenges faced by the separate healthcare systems operating within the U.S. [17], or the implications of their differing models of pain care for individuals receiving care across these systems [18].

In sum, achieving safe and effective pain management that includes opioids while mitigating the risks associated with opioids remains a significant challenge across the fragmented U.S. healthcare system. There is a need to identify core challenges and develop a focused agenda for safe and appropriate access to opioids within and across diverse healthcare systems. To address this gap, we conducted a qualitative study with a panel of national experts. Our objectives were to: (a) identify central challenges in opioid prescribing and risk mitigation; and (b) determine how best to support providers in operationalizing safe and appropriate opioid prescribing.

## Methods

Semi-structured in-depth qualitative panel discussions were conducted with healthcare providers, pain and substance use clinical and health services researchers, and individuals with a policy-making or other operational leadership role (henceforth: policy makers) working within MHS, VA, and civilian environments. Expert panel discussions were selected as an effective method for learning from participants with expertise in a highly-specialized topic area [19,20]. Expert panel discussions are recommended as a qualitative method when existing scientific evidence is complex (e.g., mixed for/against a specific therapy) and/or requires substantial synthesis [19,21]. Given that opioid prescribing in the U.S. occurs across multiple healthcare systems and clinical settings (e.g., primary care, emergency department), panel discussions were determined to be an appropriate method for gathering and synthesis of diverse perspectives. Panel discussions were conducted as part of a larger evaluation of novel opioid risk mitigation tools to identify unhealthy opioid use and unsafe clinician prescribing patterns in the MHS [10].

### Participant recruitment

Experts were purposively sampled to represent MHS, VA, and civilian clinician, healthcare policy, and research perspectives on opioid risk mitigation. Research specialties included pharmacology, mental and behavioral health services, and clinical addiction. Clinical specialties included pharmacy, preventive medicine, family medicine, primary care, emergency medicine and pain medicine; all experts were selected for their national reputation and using criteria appropriate to their respective specialties (e.g., all researchers had multiple first-author publications on relevant topics; policy makers held high-level leadership positions within their institutions; clinicians were active in clinical care as well as professional societies for their specialties). Each expert was contacted by email and asked to participate in two live video panels (90 minutes each) hosted on a web-based platform, Adobe Connect, compatible with MHS and VA regulations. Of the 32 experts contacted, 18 agreed to participate (56.3%). Those who agreed to participate were offered $200 in compensation per session.

### Data collection strategies

We asked each participant to attend two separate expert panels in an effort to ensure sufficient opportunity for in-depth discussion. In order to facilitate participation by busy experts, we offered group discussions at six different times. Fourteen participants were able to attend two of the scheduled sessions; the remaining four experts were accommodated in individual sessions, for a total of 18 participants. Expert panel discussions were conducted via Adobe Connect in May 2017, and led by a PhD-level anthropologist or clinical psychologist with expertise in qualitative methodology. A semi-structured discussion guide was developed to elucidate perspectives on: (a) challenges in opioid risk mitigation (sample question: “what do you see as the biggest challenges for providers in prescribing opioids as part of their practice?”); and (b) how best to support providers in safe and appropriate opioid prescribing across MHS, VA, and civilian health systems (sample question: “what do providers need to ensure safe and appropriate opioid prescribing for their patients?”). Participants were informed that discussions were confidential and would be recorded (audio/visual); verbal consent was documented as part of recorded discussions. The UT Health San Antonio Institutional Review Board approved all study procedures.

### Analysis

A team-based qualitative content analysis approach was used, with key themes emerging and refined during review, synthesis, and coding of discussion transcripts [22]. Panel and individual discussions were transcribed verbatim by a professional service (GMR Transcription) and uploaded to ATLAS.ti 7.0 [23] qualitative software. Three team members reviewed transcripts to identify initial themes related to challenges and recommendations. A coding manual was developed for the purpose of assigning categories (codes) to text reflecting these themes, and refined as coding proceeded. Two members of the research team (MC, SS) conducted independent coding of all transcripts, meeting weekly with a third member (EF) to identify coding disagreements, which were resolved by discussion to consensus. Individual themes and larger content areas (domains) were refined in team discussions. Themes were assigned to domains as appropriate. Following coding, the content of coded text was reviewed and synthesized. All team members participated in refining the final summation of themes.

## Results

**Fig 1:**
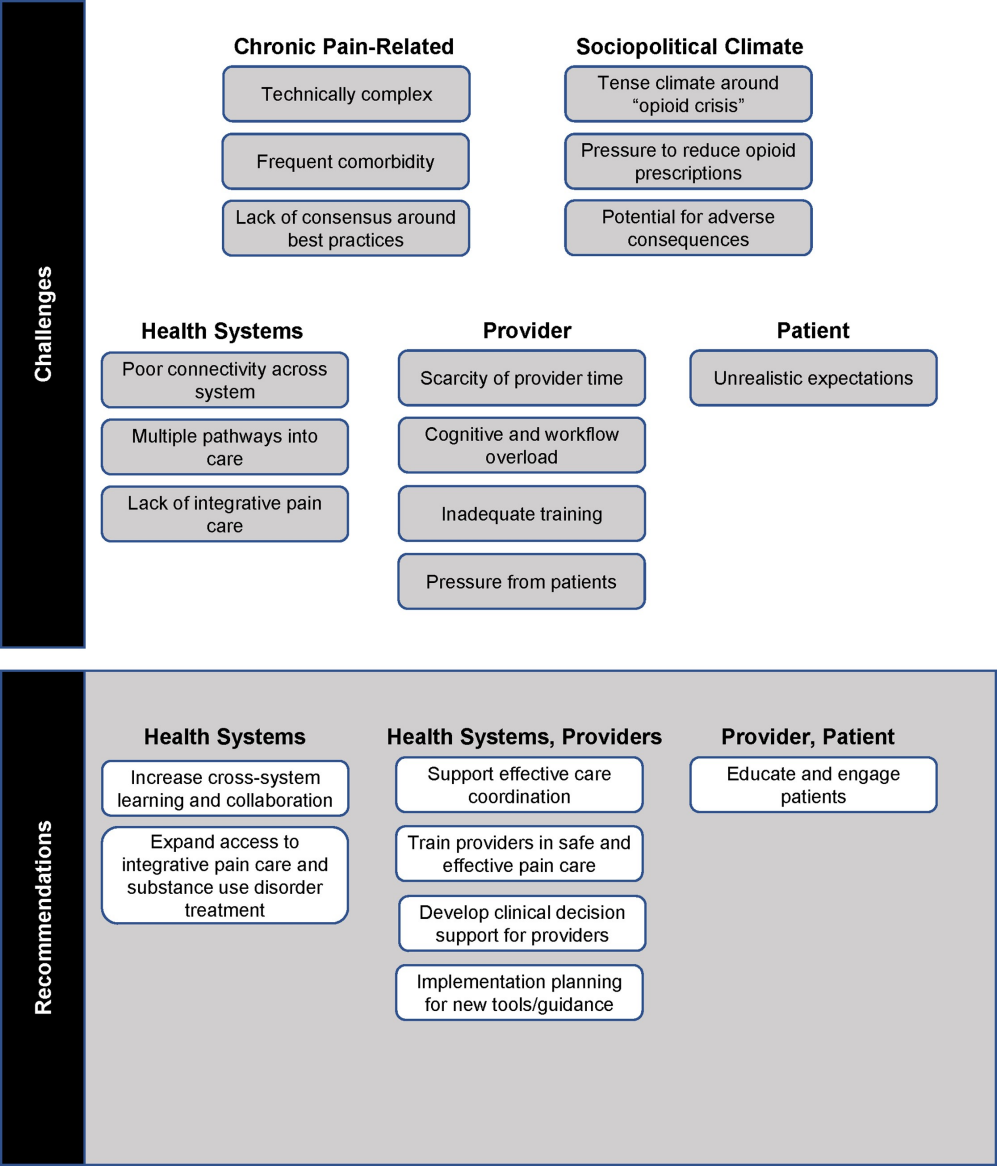
Core challenges and recommendations from national experts toward increasing the safety of opioid prescribing for chronic pain.

We conducted six semi-structured qualitative discussions with 18 individuals (Table 1). Participants represented differing roles (e.g., healthcare, policy, research) and sectors (military, VA, civilian). Most participants were male (72.2%), worked in the civilian sector (61.6%), and engaged in clinical care (55.6%; e.g., primary care, emergency medicine, pain medicine, clinical psychology, pharmacy). Expert panelists described multifactorial challenges and recommendations, as summarized below (Fig 1).

**Table 1:** Participant sample characteristics (N = 18)*.

	n (%)
Gender	
Male	13 (72.2%)
Female	5 (27.8%)
Professional Role	
Clinician	10 (55.6%)
Research	10 (55.6%)
Policy	8 (44.4%)
Health System	
Military	5 (27.8%)
VA	5 (27.8%)
Civilian	11 (61.1%)
Clinical Specialties	
Primary Care	4 (22.2%)
Chronic Pain (including clinical psychology, physical medicine & rehabilitation, emergency medicine, pharmacy)	5 (27.8%)
Addiction	3 (16.7%)
Credentials	
MD or PA	8 (44.4%)
PhD or ScD	9 (50%)
PharmD	2 (22.2%)

* participants are not mutually exclusive because often represented multiple roles; VA = Veterans Affairs; participants often had multiple credentials

### Challenges

Challenges in chronic pain management and opioid risk mitigation fell within five overarching domains: chronic pain-related challenges; sociopolitical climate; health systems; providers; and patients (summarized in Table 2).

**Table 2:** Multifactorial challenges in chronic pain management and opioid risk mitigation identified by expert panelists.

Challenges	Exemplar Quotes
Domain 1: Chronic Pain-Related Challenges	
*Chronic pain management is technically complex*.	I have often times seen patients placed on opioids for problems they won’t likely respond or certainly opioids should not be used long-term. . . .I think a lot of physicians, a lot of prescribers, struggle between the differences between the opioids. So just because patients do not respond to one opioid does not mean they wouldn’t be a candidate for an opioid. [Civilian and VA Clinician and Researcher]
*Chronic pain is frequently comorbid with other conditions*, *including mental health or substance use disorders*, *raising challenges for risk assessment and polypharmacy*.	[I]t’s hard delineating chronic pain versus addiction. I don’t know if I ever get to the right answer because I don’t think that’s always possible to sort that out in the acute care setting. [Civilian Clinician]
	[P]roviders often have patients on opioids and benzos. And the big challenge for them, is, “How do I take my patient off benzos they have been on it for 15 years? Patient really needs an opioid but I can’t leave them on a benzo.” [Civilian and VA Clinician and Researcher]
*There is a lack of consensus around evidence-based strategies for effective opioid risk mitigation*.	So, if you think that it …it is never right, or almost never right to prescribe something, then the standard of care could be…no new starts….So, there’s this underlying reality–and we don’t have the data, really, to fully resolve that. I mean, you can adopt a belief based on limited data, but it’s kind of, in the end, a little bit of hand-waving and “Well, I wind up over here.” [VA Clinician and Researcher]
Domain 2: Sociopolitical Climate as a Challenge	
*The “opioid crisis” has created a climate of tension around opioid prescribing*.	So we have to look at providers because they are stuck in this quagmire of media frenzy and this challenge to reduce opioid use. [Civilian and VA Clinician and Researcher]
*Providers feel pressure to rapidly reduce the number and dose of opioid prescriptions for patients with chronic pain*.	There’s a lot of pushback against physicians from their leadership and also just from, uh, they–our state organizations, national organizations to avoid using these things, and it’s not worth losing your livelihood… [Civilian Clinician]
*There is significant concern about negative consequences of rapid reduction in opioid availability*.	I’m always concerned because I know there are some providers who are going to respond to this in a way that is not so good for patients, who are going to say, “I’m just going to stop prescribing, everybody is going to be tapered to this dose,” and those sorts of things. [Civilian Clinician and Policy]
Domain 3: Health Systems-Level Challenges	
*Poor interconnectivity between electronic medical records and other data monitoring systems results in gaps in data and creates burden for providers*.	There is a push here… to make it mandatory, to query the PDMP for all patients before you prescribe an opioid or administer an opioid. And there is push back from various advocacy groups …because they feel like it takes too much time. [Military Clinician]
*Chronic pain patients may enter a system through a variety of specialties*, *raising challenges for care coordination*, *follow-up*, *and identification of potential risk or misuse*.	If the clinician is looking at a prescription pad and there’s not a mixture of physical therapy and maybe a pain psychologist and a bona fide mental-health provider for the patient’s incipient PTSD, then that–all of that becomes very high-risk situation. [VA Clinician and Researcher]
*Access to high-quality pain management is lacking for many patients*, *as a result of poor healthcare coverage*, *a shortage of specialty providers*, *and costs of care*.	I think a big challenge for clinicians is they don’t have access to behavioral health clinicians. That’s huge! They get in over their head, they need that help. I couldn’t function at my clinic if I didn’t have a psychologist there. [Civilian and VA Clinician and Researcher]
	[I]f the clinician is looking at a prescription pad and there’s not a mixture of physical therapy and, uh, maybe a pain psychologist and a–a bona fide mental-health provider for the patient’s incipient PTSD, then that–all of that becomes [a] very high-risk situation. [VA Clinician and Researcher]
Domain 4: Provider-Level Challenges	
*Provider time for clinical care is a scarce resource*.	I would tell you that the primary care provider, has a 20-minute appointment that they can see these patients for. If they’re a pain specialist they can [take] 30 and sometimes more. Emergency doctors… a lot of times, that 20 minutes includes the intake. So, they might get 12–15 minutes at the most if they’ve got a good technician to get these patients. So, they’ve got to see them–most of these visits aren’t 15 minutes or 20 minutes. [Military Clinician]
	[I]f you’re cutting costs and cutting corners by having less staff, less front office, less medical assistants, nobody to scribe to write your progress notes, you’re just in there running around like a chicken with your head cut off. [Civilian Clinician]
*Many providers are overloaded by workflow and information demands*.	Adding another tool for…is going to create another bucket that we’ll have to check every time. Providers are in such overload they will not do that. [Military Clinician]
*Many providers are inadequately trained to deliver effective chronic pain care (e*.*g*., *patient risk assessment*, *treatment planning*, *use of information resources*, *and communication with patients around pain management)*.	[A] lot of clinicians say they struggle with how to have the conversations with the patients that they’re concerned about and struggling with. And just having the data doesn’t give them that training. [VA Clinician and Researcher]
	[W]e have a lot of junior docs or mid-level practitioners that may not have enough training in MME to kind of make those decisions, yet they still have the ability to prescribe opiates. [Military Clinician]
*Providers may face pressure from patients to provide opioid medications*.	Many of the patients have, um, uh, the–they developed that antagonistic attitude that the opiate is the metric by which they judge how well they’re being treated. [Civilian Clinician]
	…[T]he opioid epidemic is getting worse and worse because people are willing to just prescribe opioids to bump their patient satisfaction scores higher, without any sort of aggressive patient tailoring. . . [Civilian Clinician]
Domain 5: Patient-Level Challenges	
*Patients may have unrealistic expectations regarding pain management*.	[O]ne of the things that might also be helpful…is also helping patients understand and get their expectations in place….Because I think [for] a lot of patients… what was nice about opioids you just give them a pill. This [cognitive behavioral therapy] it may take a few sessions or 3 or 4 weeks for you to maybe see some of these effects. But I think we need to really set those expectations up and help them understand their options. And then figure out, work with them to help them understand what is acceptable for them kinda going forward. [VA Researcher and Policymaker]

#### Chronic pain-related challenges

*Chronic pain management is technically complex*. Panel participants noted that delivery of effective care for chronic pain requires in-depth knowledge of patient assessment, appropriate medications and dosing, potential drug interactions, behavioral health and other non-pharmacological treatments, and recommended guidelines for care. They acknowledged that delivering these treatments in real time could pose a challenge.

There’s this reality that all the decisions that go forward require a very individualized, patient-centered aspect, and we have trouble operationalizing that. We’re trying, obviously, but it’s tough. [VA Clinician and Researcher]

*Chronic pain is frequently comorbid with other conditions*. Including mental health or substance use disorders. This increases the complexity of assessing patient needs, risk for misuse, treatment options, and potential for interaction with other medications.

I think the biggest [challenges] are related to patients who have high comorbidity, patients with identified or undetected mental health, psychological problems, or substance use disorders… how do you know that this is [a] patient that might have some problems in the future? [Civilian Research and Policy]

*There is a lack of consensus around evidence-based strategies for effective chronic pain management and opioid risk mitigation*. Panelists cited a lack of evidence regarding which treatments are safe and effective for chronic pain, what types of patients and conditions are most likely to benefit from opioids or other treatments, and ambiguity in how best to measure treatment benefits. One panelist stated that, “We don’t have a good way to treat pain.” Another panelist cited evidence that a patient’s prior mental health and substance use history can serve as an important signal for opioid misuse potential; however, the same expert noted the evidence is not entirely clear and may serve as a “green light for [offering opioids to] the low-risk patient.” Panelists also noted that, while there is significant work ongoing to develop tools and strategies in support of safe and appropriate chronic pain management, many of the existing strategies lack a strong evidence base.

The strategies are put out there to reduce risks–they are risk stratification and monitoring so you… identify whether patients have risk factors to becoming addicted to opioids…you monitor them closely, so more frequent urine drug testing, you would do more frequent follow up visits, you’d have treatment agreements–all that sort of stuff. There’s no… all the things are sensible and good… but there’s no evidence that they reduce risk. [Civilian Research and Policy]

#### Sociopolitical climate as a challenge

*At the national level*, *the “opioid crisis” has created a climate of tension around opioid prescribing*. Panelists described a national climate in which policy discussions about effective strategies for managing the crisis may be shaped by the emotional tenor of public debate, and in which even informational tools and resources may be “weaponized” and used to penalize patients and/or providers. They delineated a history of rapid change in the expected standard of care for patients with chronic pain, detailing a shift from heavy reliance on long-term opioids to significant concern about any use of opioids on a long-term basis.

So I think the biggest challenge is a lot of the political rhetoric around opioid use. No question opioids are dangerous but I think it has gotten way blown out of proportion compared to other medication, compared to other issues. NSAIDS for example have a very high risk of death from a coronary heart disease, hospitalizations, kidney dysfunctions, dialyses. Not to minimize the risk with opioids but we don’t want to forget many other drugs have issues. [Civilian and VA Clinician and Research]

*Providers feel pressured to rapidly reduce the number and dose of opioid prescriptions for patients with chronic pain*. In response to concerns about opioid prescribing, there has been a rapid swing within many healthcare organizations toward reducing the number of new prescriptions, and toward reducing the number and dose of long-term opioid prescriptions among existing patients with chronic pain. Providers may end up feeling “stuck in this quagmire of media frenzy and this challenge to reduce opioid use,” with implications for how they approach prescribing:

I’m always concerned because I know there are some providers who are going to respond to this in a way that is not so good for patients, who are going to say, I’m just going to stop prescribing, everybody is going to be tapered to this dose, and those sorts of things.[Civilian Clinician and Policymaker]

*There is significant concern about negative consequences of rapid reduction in opioid availability*. Panelists described fears that the rapid shift in prescribing practice was resulting in a lack of patient-centered care and potential harm for patients. Panelists working across sectors perceived significant danger in failing to provide effective treatment for patients who had received long-term opioids for chronic pain, in particular describing the risks of leaving patients to “search for solutions on their own,” with the potential for opioid misuse, use of illegal substances, addiction, overdose, or suicide.

I’ve observed a real rush to make the opioid numbers go down, and in doing so, I’ve personally attended to a large number of patients who’ve been directly harmed by involuntary tapers and discontinuations… [VA Clinician and Researcher]

#### Health systems-level challenges

*Poor interconnectivity between electronic medical records and other data monitoring systems results in gaps in data and creates burden for providers*. Patient healthcare often occurs across multiple systems (e.g., military and civilian), with the result that data on opioid prescribing may be incomplete and difficult to access. Although state-based PDMPs gather pharmacy data on controlled prescriptions, experts reported that neither VA nor MHS health systems were consistently reporting to the PDMP, limiting their utility and requiring providers to run multiple queries. Barriers to PDMP use (e.g., complicated login) can increase the time required for each query of available prescribing data, while concerns about the quality of available data may reduce the perceived value of such queries.

I end up querying the PDMP for everybody. And for most of my patients, I’m pulling up their past medical history in the electronic medical record anyway, so it’s an additional two to five minutes of work for every patient that comes in… [Military Clinician]

*Chronic pain patients may enter a system through a variety of specialties*, *e*.*g*., *primary care*, *emergency medicine*, *or mental health*. Panelists noted that this raises additional challenges for care coordination, follow-up, and identification of potential opioid misuse.

What I see is people are being referred to mental health, “Oh, it’s addiction so we are going to refer them to mental health,” and then mental health is like, “Oh, pain is related to opioids—that’s not my thing, I am gonna refer them back to the primary care doc.” So we are having a lot of patients left in limbo …. Because ideally we manage them where they show up, right? … [VA Researcher and Policymaker]

*Access to high-quality chronic pain management is lacking for many patients*. Panelists also reported that gaps in healthcare coverage and access impact the delivery of chronic pain care nationally, citing a lack of available specialty providers, particularly in behavioral health, lack of reimbursement available for non-pharmacological pain care, and lack of resources to support providers in “humanely and appropriately” weaning patients off opioids when needed.

[A] lot of people have drunk the Kool-Aid that we need to treat pain differently and there has been a big emphasis on alternatives to opioids, and in psychology, behavioral medicine, [as] an obvious component of that. The problem is that patients don’t have great access to the treatments that we know are evidence-based, that we know work, that doctors would like to plug their patients into. This is the fundamental problem in my mind…[Civilian and VA Clinician and Research]

#### Provider-level challenges

*Provider time for clinical care is a scarce resource*. Despite general agreement that delivering effective, patient-centered care for patients with chronic pain requires time and attention, panelists noted that a lack of time remains a core challenge at the provider level.

The issue that you’re going to see regardless though is time is kind of the capital of the primary care clinic…. That conversation [about pain management] takes time, so it’s really going to be a big cultural change to less spectator medicine and more proactive, you know, getting in there and fixing these problems, which can be very hard to do in certain clinics. [Military Clinician]

*Many providers are overloaded by workflow and information demands*. Knowledge and technologies to support high-quality chronic pain care are available and proliferating; however, panelists noted that the utility of existing tools to support quality pain care is limited by providers’ cognitive overload.

One of the issues we deal with, day in and day out, is all the “good idea” fairies that add

to the primary care doc’s load…I have to be aware of not just this good idea, but the good idea about what language they want to use and what their healthcare literacy score is and whether they’re safe at home…[I]t’s just–it’s a bit overwhelming, the number of things that go on a primary care doc’s plate. [Civilian and Military Policy]

*Many providers are inadequately trained to deliver effective chronic pain care*. Given the complexity of chronic pain care, panelists frequently noted the lack of provider training in skills such as patient opioid risk assessment, treatment planning, use of informational resources, and communication with patients around pain management.

[I]t is going to be difficult with the pendulum swinging back to figure out how to taper, when a lot of primary care providers don’t know how to do that and how to do that safely and in a patient-centered fashion. [VA Research and Policy]

[S]ome [providers] are more uncomfortable than others with the tough conversations that can come with…using opiates in your treatment practices… [VA Clinician and Research]

*Providers may face pressure from patients to provide opioid medications*. Panelists also pointed out that patients with chronic pain may actively request an opioid prescription, and may be unsatisfied with a provider who refuses this request.

[I]f I’m seeing a patient who has already developed a strong idea that opioids are somehow the–either helpful or the token by which they judge whether their doctor is engaged with them, so, “You provide this to me or I’m gonna accuse you of being not committed to me.” [VA Clinician and Researcher]

In addition to having to cope with an unhappy patient, providers in some settings of care may be penalized if they receive low ratings on patient satisfaction related to a reluctance to prescribe opioids.

Patient satisfaction is directly tied to do you receive pain medication, opioid specifically, and do you receive antibiotics….[I]n the civilian world that’s directly tied to your ability to be paid to your maximum amount and to keep your job even. [Military Clinician]

#### Patient-level challenges

*Patients may have unrealistic expectations regarding chronic pain management*. At the level of the patient, panelists pointed to the fact that many patients seeking care for chronic pain do so with the expectation that medication is the best treatment option, or that effective care will achieve immediate results or result in a life without pain.

[O]ne of the things that is the most difficult issue to deal with, with patients who come in with a complaint of pain is managing their expectations. Some people are just not going to get complete pain relief, and they are expecting to be perfect. [Civilian Clinician]

### Recommendations

Recommendations outlined by panelists are described below (see also Table 3), beginning at the systems level and narrowing to those targeting providers and/or patients.

**Table 3:** Recommendations for achieving safe and effective chronic pain management and opioid risk mitigation identified by expert panelists.

Recommendations	Exemplar Quotes	Domains Involved
*Increase cross-system learning and collaboration*.	We [MHS] work very closely with the VA, and we’ve been drafting off a lot of their successes and I don’t call them failures. Even times where the VA has had a rough go of it, they’ve led the way in a lot of the things that the [MHS] is benefiting by…they’ve shared a lot of experience with us. [Military Policymaker]	Health System
*Expand access to integrative chronic pain care and treatment for substance use disorders*.	…[T]here really does need to be some kind of team based multi-modal care available for these patients, especially reactivation and cognitive behavioral therapy, even if it’s just brief interventions… [Civilian Researcher and Policymaker]	Health System
	[H]ow do we help patients gain access to the treatments that we know work? …And how do we help patients also wean down on opioids by accessing these evidence-based treatments? And this may involve expanding our thinking around how those treatments are delivered…broadening access by utilizing novel modalities may involve some online treatments and education as one example. May involve online modalities and treatments. [Civilian and VA Clinician and Researcher]	
*Support effective care coordination for patients with chronic pain*.	I just think that…having all of the prescribers and all their care providers understand what the goals are for that patient, so you don’t get contradictory, mixed messages, I think is very important for having the patient able to do well. [Civilian Clinician]	Health System, Provider
*Develop clinical decision support (CDS) for providers without increasing cognitive load*.	“Oh, great. More information.” So, if something happens to this patient, they pull this up and they say, “Dr. [So-and-so]” and they bring [him or her] in and say, “didn’t you see this? Didn’t you look at all this stuff and don’t you realize how dangerous it was for you to give this patient another oxycodone?” So, you’re saying, “this is all the help you got” and I’m all saying, “Oh, man. That lawyer’s going to nail me eventually because all this was available and I didn’t take the time to go through it.” [Civilian and Military Policy]	Health System, Provider
*Implement new measures (tools*, *resources) with appropriate training and support (e*.*g*. *clinical decision support)*.	As far as something actionable. . .some healthcare leader in that institution needs to be involved in this. This will need consistent reinforcement, otherwise you’re going to get folks who do this great for about a month or two, and then they’re like, “Ahh it’s too much work, I’m done,” and then they never see it again. So you have to have that sustainment…that continued implementation tail. [Military Clinician]	Health System, Provider
*Train providers in safe and effective pain care*, *to include application of clinical practice guidelines*, *use of information resources and risk management practices (e*.*g*., *urine drug screens*, *opioid tapering) and communication with patients (e*.*g*., *realistic goal-setting*, *shared decision-making)*.	[H]ow do you link [providers] to the kind of continuing medical education that is going to help them make the changes they want to make? [Civilian Clinician and Policymaker]	Health System, Provider
	[T]he physicians need skills in talking to patients and listening to them so you can help patients with chronic pain with just listening to them and reinforcing things that they’re doing that may be helpful. [Civilian Researcher and Policymaker]	
*Educate and engage patients in safe and effective pain management*.	[H]elping patients understand and get their expectations in place. Knowing that ok, a lot of people are like, “Oh well I tried yoga and it didn’t work,” or “I tried massage and physical therapy and it didn’t work.” But I think like getting their expectations in place like you may need to try this out for x amount of time for clinical effectiveness. …But I think we need to really set those expectations up and help them understand their options. And then figure out, work with them to help them understand what is acceptable for them kinda going forward. [VA Researcher and Policymaker]	Patient, Provider

#### Increase cross-system learning and collaboration

Panelists noted that the recent crisis within pain care has been accompanied by a surge in the available knowledge and resources for chronic pain management across VA, military, and civilian health systems. This growth provides an opportunity to engage in cross-system learning and collaboration:

We [MHS] work very closely with the VA, and we’ve been drafting off a lot of their successes and I don’t call them failures. Even times where the VA has had a rough go of it, they’ve led the way in a lot of the things that the [MHS] is benefitting by…they’ve shared a lot of experience with us. [Military Policymaker]

#### Expand access to integrative chronic pain care and treatment for substance use disorders

In response to access gaps described above, panelists emphasized the importance of increasing availability of services for integrative chronic pain care and substance use disorder treatment.

[F]rom our perspective and experience, it’s a matter of having a menu of options that are non-opioid, non-medication, available. So when you do choose opioids, it’s appropriate and required, and not just because there’s nothing else to offer the patient. [Military Policymaker]

#### Support effective care coordination for patients with chronic pain

With the benefits of making multi-modal pain treatment more available comes enhanced need for communication and follow-up with patients and within the care team. Panelists noted that such care coordination requires appropriate staffing, shared goals, and outcomes monitoring:

…[T]hey probably need an invested and supportive non-prescriber staff, most notably nurses, to ascertain if the care and the patient are kind of going according to plan. [VA Clinician and Researcher]

#### Develop clinical decision support (CDS) for providers without increasing cognitive load

Panelists discussed at length what kinds of tools and resources would be of greatest utility for providers, generally preferring CDS that support effective practice in chronic pain and opioid risk assessment, treatment planning, and outcomes and safety monitoring. Panelists also offered the caveat that more information is not always better, particularly if it increases demand on providers’ time and attention, and emphasized the importance of making tools simple and functional:

…I would suggest giving people tools to help with [opioid management] and not overwhelming them…. very nice, simple, provider-focused tools that are meant for really busy providers to be able to do that. [VA Researcher and Policymaker]

#### Train providers in safe and effective chronic pain care

Panelists noted that providers across an array of specialties provide care for acute and/or chronic pain, often with minimal training. Panelists pointed to need for provider training in application of clinical practice guidelines, use of existing information resources (e.g., PDMPs, CDS) and risk management practices (e.g., urine drug screens, opioid tapering), and effective communication with patients (e.g., realistic goal-setting, shared decision-making).

[G]ive the providers that are [asking for help] some education and tools in how to address this type of patients in the future. [Civilian Clinician]

#### Implement new measures with appropriate training and support to achieve uptake and sustainment

Panelists also noted the importance of ensuring new tools (e.g., CDS), guidelines, and other resources (e.g., screening or referral technologies) are introduced alongside appropriate training and other implementation strategies, such as backing from leadership, clinical champions, and/or continuing education and feedback, in order to ensure uptake and sustainment.

[S]ome healthcare leader in that institution needs to be involved in this. This will need consistent reinforcement, otherwise you’re going to get folks who do this great for about a month or two, and then they’re like, “Ahh it’s too much work, I’m done,” and then they never see it again. So you have to have that sustainment…that continued implementation tail. [Military Clinician]

#### Educate and engage patients in safe and effective chronic pain management

Panelists offered a vision for supporting patients to achieve reduced pain intensity and improved functioning and quality of life. They cited the need for resources to help patients set and achieve realistic goals for pain management.

I think we need to really set those expectations up and help [patients] understand their options. And then figure out, work with them to help them understand what is acceptable for them kinda going forward. [VA Researcher and Policymaker]

## Discussion

We conducted qualitative discussions with a panel of national experts to identify key challenges and recommendations in reducing risks associated with prescribed opioids. Panelists provided insight into challenges across multiple levels of the U.S. health system, including: the technical complexity of treating chronic pain; the fraught national climate around opioids; the need to integrate surveillance data across a fragmented health system; a lack of access to non-pharmacological options for chronic pain care; and the difficulties inherent in asking providers and patients to negotiate treatment for a complex condition in brief clinical encounters, often without adequate knowledge on either side of the risks, benefits and/or availability of chronic pain care options. Panelists’ discussion of core challenges for providers underscore findings of other recent studies, in which providers described navigating a “tightrope” between the push to avoid opioid prescriptions for chronic pain and pressure from patients to prescribe [10,24–26]. A study of patient satisfaction scores by Sites et al. [27] found that, among patients with musculoskeletal pain, those receiving opioid medications were more likely to report being highly satisfied with their care. Patients with chronic pain, meanwhile, may report feeling reliant on opioids even when expressing ambivalence regarding their benefit [28,29]. Given this, prescribers’ sense of walking a tightrope becomes understandable, particularly given the tense national climate described by participating experts.

In the course of these expert discussions, panelists collaboratively imagined a healthcare delivery system that would ensure access to behavioral health and other non-pharmacological therapies for chronic pain, achieve interconnectivity and care coordination within and across health systems, utilize thoughtful CDS and tools to support providers in real-time assessment of likely treatment risks and benefits, and provide education and training for both providers and patients. They recommended that clinical teams would also facilitate patients’ access to consultation and treatment for substance use disorders [30], while permitting opioid use as part of safe and appropriate pain management for those most likely to benefit. Establishment of the new Defense Health Agency may enhance opportunities to improve collaboration across the MHS, civilian sector, and VA.

These findings point to challenges and opportunities at multiple levels, beginning with providers and patients themselves. Repeated studies have shown deficits in patient-provider communication around opioid prescribing for acute and chronic pain [31–33], consistent with those described by panel members. These deficits persist despite availability of a variety of resources to educate providers to engage in “healthy dialogue” with patients around appropriate use of opioids [34]. Participating experts also highlighted continued need to identify and disseminate evidence-based practices for comprehensive pain management, to include best practices for opioid prescribing and screening and referral for substance use disorders, as needed. Although there is growing consensus regarding recommendations for chronic pain care [6], opioid risk mitigation strategies remain complex. Providers require ongoing training and support to implement recommendations. Practical tools for decision support in busy clinical settings[10,35] are required, but must be feasible for health care providers to use routinely amid juggling a continuum of other health issues.

At the broader system level, panelists acknowledged that access to both nonpharmacologic pain care and substance use disorder treatment remains constrained in many–and particularly rural–areas. There are well-recognized disparities in the availability of specialty providers and evidence-based treatment options across the country [36–40]. In addition, many healthcare insurance plans limit coverage for nonpharmacologic chronic pain treatment options, despite efforts to increase parity in coverage of mental health and substance use care under the Affordable Care Act [41–43]. Healthcare coverage plans may also be inconsistent with CDC and other guidelines for pain care, which explicitly recommend multimodal pain care and integration of CBT. Although one survey of state Medicaid agencies found that most reported providing at least some coverage for non-opioid pain treatment [44], a cross-sectional study of 45 plans representing Medicaid, commercial, and Medicare Advantage insurers found that few covered acupuncture or psychological interventions like CBT for chronic non-cancer pain [45]. Evidence from the VA suggests, however, that patients are receptive to nonpharmacologic pain management when those options are made available [44]. Thus efforts to ensure healthcare coverage aligns with guidelines for effective pain care are likely to remain an important area for future work. Creative solutions must also be identified to address geographic impediments to comprehensive pain management.

The method of qualitative panel discussions used here has a number of strengths, which include reflecting expert knowledge and incorporating diverse national, cross-system, and multidisciplinary perspectives [46]. Given constraints on expert panelists’ time, we did not adopt a formalized process of developing consensus (e.g., Delphi method); as a result, weighting recommendations in terms of their perceived importance or feasibility awaits further investigation. Our semi-structured discussion approach, however, allowed for more in-depth exploration of topics than is often possible with consensus-focused methods [47]. Additional limitations included modest sample size and incomplete representation across diverse medical specialties and health systems.

These findings point to significant challenges facing the U.S. health system as opioid overdose remains a leading cause of injury and death [48,49]. Leaders and policymakers will need to weigh the needs and challenges specific to their own agencies and institutions, and select appropriate next steps in light of existing initiatives, perceived needs, and available resources. VA, for example, has implemented several of the recommendations listed here as part of its Opioid Safety Initiative and other programs, and has seen a corresponding drop in high-dose opioid prescribing among Veterans [50]. As of late 2017, VA was required to report to state PDMPs, thereby increasing the accessibility of prescribing data across VA and non-VA systems [51]. Despite this progress, continued reductions in opioid-related morbidity and mortality are likely to require a public health perspective and broader collaboration across our nation’s healthcare systems to identify gaps and “loopholes” in chronic pain care, and to develop targeted, patient-centered solutions for opioid risk mitigation [52].

Significant federal resources have been brought to bear on prevention, treatment and recovery from opioid use disorder. However, without addressing these and other issues, we risk failing to make the fundamental changes in our health care system that will prevent the next opioid ‘crisis’.

## References

[1] Chou R, Turner JA, Devine EB, Hansen RN, Sullivan SD, Blazina I, et al The Effectiveness and Risks of Long-Term Opioid Therapy for Chronic Pain: A Systematic Review for a National Institutes of Health Pathways to Prevention Workshop. Ann Intern Med. 2015 2 17;162(4):276 10.7326/M14-2559 25581257

[2] Baldini A, Von Korff M, Lin EHB. A Review of Potential Adverse Effects of Long-Term Opioid Therapy: A Practitioner’s Guide. Prim Care Companion CNS Disord [Internet]. 2012 6 14 [cited 2018 May 14]; Available from: http://www.psychiatrist.com/pcc/article/pages/2012/v14n03/11m01326.aspx 10.4088/PCC.11m01326 PMC3466038 23106029

[3] Volkow ND, McLellan AT. Opioid Abuse in Chronic Pain—Misconceptions and Mitigation Strategies. Longo DL, editor. N Engl J Med. 2016 3 31;374(13):1253–63. 10.1056/NEJMra1507771 27028915

[4] Weisberg DF, Becker WC, Fiellin DA, Stannard C. Prescription opioid misuse in the United States and the United Kingdom: Cautionary lessons. Int J Drug Policy. 2014 11;25(6):1124–30. 10.1016/j.drugpo.2014.07.009 25190034

[5] García MC, Dodek AB, Kowalski T, Fallon J, Lee SH, Iademarco MF, et al Declines in Opioid Prescribing After a Private Insurer Policy Change—Massachusetts, 2011–2015. MMWR Morb Mortal Wkly Rep. 2016 10 21;65(41):1125–31. 10.15585/mmwr.mm6541a1 27764082

[6] Dowell D, Haegerich TM, Chou R. CDC Guideline for Prescribing Opioids for Chronic Pain—United States, 2016. MMWR Recomm Rep. 2016 3 18;65(1):1–49. 10.15585/mmwr.rr6501e1 26987082

[7] VA/DoD Clinical Practice Guideline for Management of Opioid Therapy for Chronic Pain [Internet]. The Management of Opioid Therapy for Chronic Pain Working Group, Department of Veterans Affairs, Department of Defense.; 2010 [cited 2018 May 14]. Available from: https://www.va.gov/painmanagement/docs/cpg_opioidtherapy_fulltext.pdf

[8] Finley EP, Garcia, Ashley, Rosen, Kristen, McGeary, et al Evaluating the Impact of Prescription Drug Monitoring Programs: A Scoping Review. BMC Health Serv Res. 2017;17(1):420 10.1186/s12913-017-2354-5 28633638 PMC5477729

[9] Gudin J. Risk Evaluation and Mitigation Strategies (REMS) for Extended-Release and Long-Acting Opioid Analgesics: Considerations for Palliative Care Practice. J Pain Palliat Care Pharmacother. 2012 6 22;26(2):136–43. 10.3109/15360288.2012.679724 22764852

[10] Finley EP, Schneegans S, Tami C, Pugh MJ, McGeary D, Penney L, et al Implementing prescription drug monitoring and other clinical decision support for opioid risk mitigation in a military health care setting: a qualitative feasibility study. J Am Med Inform Assoc JAMIA. 2018 01;25(5):515–22. 10.1093/jamia/ocx075 29025024 PMC7646964

[11] Kazanis W, Pugh MJ, Tami C, Maddry JK, Bebarta VS, Finley EP, et al Opioid Use Patterns Among Active Duty Service Members and Civilians: 2006–2014. Mil Med. 2018 3 1;183(3–4):e157–64. 10.1093/milmed/usx014 29514335 PMC6927844

[12] Centers for Disease Control and Prevention. 2019 Annual Surveillance Report of Drug-Related Risks and Outcomes—United States Surveillance Special Report. Centers for Disease Control and Prevention, U.S. Department of Health and Human Services. [Internet]. 2019 p. 128. Available from: https://www.cdc.gov/drugoverdose/pdf/pubs/2019-cdc-drug-surveillancereport.pdf

[13] Volkow N, Benveniste H, McLellan AT. Use and Misuse of Opioids in Chronic Pain. Annu Rev Med. 2018 1 29;69(1):451–65. 29029586 10.1146/annurev-med-011817-044739

[14] Eibner C, Krull H, Brown KM, Cefalu M, Mulcahy AW, Pollard M, et al Current and Projected Characteristics and Unique Health Care Needs of the Patient Population Served by the Department of Veterans Affairs. Rand Health Q [Internet]. 2016 5 9 [cited 2020 Jan 21];5(4). Available from: https://www.ncbi.nlm.nih.gov/pmc/articles/PMC5158228/10.7249/rhq-05-04-13 PMC5158228 28083423

[15] Moore M, Wermuth MA, Cecchine G, Colthirst P. Enhancing Military–Civilian Medical Synergies: The Role of Army Medical Practice in Civilian Facilities [Internet]. 2016 [cited 2019 Dec 19]. Available from: https://www.rand.org/pubs/research_reports/RR1313.html 10.7249/rhq-06-02-08 PMC5568162 28845346

[16] Mundell BF, Friedberg MW, Eibner C, Mundell WC. US Military Primary Care: Problems, Solutions, And Implications For Civilian Medicine. Health Aff (Millwood). 2013 11 1;32(11):1949–55. 10.1377/hlthaff.2013.0586 24191085

[17] Institute of Medicine (US) Committee on Assuring the Health of the Public in the 21st Century. The Health Care Delivery System. In: The Future of the Public’s Health in the 21st Century [Internet]. National Academies Press (US); 2002 [cited 2020 Jan 21]. Available from: https://www.ncbi.nlm.nih.gov/books/NBK221227/25057638

[18] Institute of Medicine (US) Committee on Advancing Pain Research, Care, and Education. Care of People with Pain. In: Relieving Pain in America: A Blueprint for Transforming Prevention, Care, Education, and Research [Internet]. Washington (DC): National Academies Press (US); 2011 [cited 2019 Dec 19]. Available from: https://www.ncbi.nlm.nih.gov/books/NBK92517/22553896

[19] Waltz TJ, Powell BJ, Matthieu MM, Chinman MJ, Smith JL, Proctor EK, et al Innovative methods for using expert panels in identifying implementation strategies and obtaining recommendations for their use. Implement Sci [Internet]. 2015 12 [cited 2018 May 14];10(S1). Available from: http://implementationscience.biomedcentral.com/articles/10.1186/1748-5908-10-S1-A44

[20] Dunn RL, Kalich KA, Henning MJ, Fedrizzi R. Engaging Field-Based Professionals in a Qualitative Assessment of Barriers and Positive Contributors to Breastfeeding Using the Social Ecological Model. Matern Child Health J. 2015 1;19(1):6–16. 10.1007/s10995-014-1488-x 24740721

[21] Coulter I, Elfenbaum P, Jain S, Jonas W. SEaRCH^TM^ expert panel process: streamlining the link between evidence and practice. BMC Res Notes [Internet]. 2016 1 7 [cited 2020 Jan 21];9. Available from: https://www.ncbi.nlm.nih.gov/pmc/articles/PMC4704387/10.1186/s13104-015-1802-8 PMC4704387 26744077

[22] Hsieh H-F, Shannon SE. Three Approaches to Qualitative Content Analysis. Qual Health Res. 2005 11;15(9):1277–88. 10.1177/1049732305276687 16204405

[23] Atlas.ti 7.0 software. Berlin: Scientific Software Development;

[24] Berg KM, Arnsten JH, Sacajiu G, Karasz A. Providers’ Experiences Treating Chronic Pain Among Opioid-Dependent Drug Users. J Gen Intern Med. 2009 4 1;24(4):482–8. 10.1007/s11606-009-0908-x 19189194 PMC2659151

[25] Matthias MS, Parpart AL, Nyland KA, Huffman MA, Stubbs DL, Sargent C, et al The Patient–Provider Relationship in Chronic Pain Care: Providers’ Perspectives. Pain Med. 2010 11 1;11(11):1688–97. 10.1111/j.1526-4637.2010.00980.x 21044259

[26] Zgierska A, Miller M, Rabago D. Patient Satisfaction, Prescription Drug Abuse, and Potential Unintended Consequences. JAMA. 2012 4 4;307(13):1377–8. 10.1001/jama.2012.419 22474199 PMC3581314

[27] Sites BD, Harrison J, Herrick MD, Masaracchia MM, Beach ML, Davis MA. Prescription Opioid Use and Satisfaction With Care Among Adults With Musculoskeletal Conditions. Ann Fam Med. 2018 1;16(1):6–13. 10.1370/afm.2148 29311169 PMC5758314

[28] Simmonds Maureen, Finley Erin P., Vale Shruthi, et al A qualitative study of Veterans on long- term opioid analgesics: Barriers and facilitators to multimodality pain management. Pain Med. In press; 10.1111/pme.12626 25528887

[29] Penney LS, Ritenbaugh C, DeBar LL, Elder C, Deyo RA. Provider and patient perspectives on opioids and alternative treatments for managing chronic pain: a qualitative study. BMC Fam Pract [Internet]. 2016 12 [cited 2019 Jan 11];17(1). Available from: http://bmcfampract.biomedcentral.com/articles/10.1186/s12875-016-0566-0 10.1186/s12875-016-0566-0 PMC5390355 28403822

[30] Kolodny A, Courtwright DT, Hwang CS, Kreiner P, Eadie JL, Clark TW, et al The Prescription Opioid and Heroin Crisis: A Public Health Approach to an Epidemic of Addiction. Annu Rev Public Health. 2015 3 18;36(1):559–74. 25581144 10.1146/annurev-publhealth-031914-122957

[31] Smith RJ, Rhodes K, Paciotti B, Kelly S, Perrone J, Meisel ZF. Patient Perspectives of Acute Pain Management in the Era of the Opioid Epidemic. Ann Emerg Med. 2015 9 1;66(3):246–252.e1. 10.1016/j.annemergmed.2015.03.025 25865093

[32] Hughes HK, Korthuis PT, Saha S, Eggly S, Sharp V, Cohn J, et al A mixed methods study of patient–provider communication about opioid analgesics. Patient Educ Couns. 2015 4 1;98(4):453–61. 10.1016/j.pec.2014.12.003 25601279 PMC4417607

[33] Matthias MS, Krebs EE, Bergman AA, Coffing JM, Bair MJ. Communicating about opioids for chronic pain: A qualitative study of patient attributions and the influence of the patient–physician relationship. Eur J Pain. 2014;18(6):835–43. 10.1002/j.1532-2149.2013.00426.x 24921073

[34] Center for Disease Control & Prevention. Applying CDC’s Guideline for Prescribing Opioids: Module 3: Communicating with Patients [Internet]. [cited 2020 Jan 21]. Available from: https://www.cdc.gov/drugoverdose/training/communicating/accessible/training.html

[35] Trafton JA, Martins SB, Michel MC, Wang D, Tu SW, Clark DJ, et al Designing an automated clinical decision support system to match clinical practice guidelines for opioid therapy for chronic pain. Implement Sci. 2010 4 12;5(1):26. 20385018 10.1186/1748-5908-5-26 PMC2868045

[36] Cummings JR, Allen L, Clennon J, Ji X, Druss BG. Geographic access to specialty mental health care across high- and low-income U.S. communities. JAMA Psychiatry. 2017 5 1;74(5):476–84. 10.1001/jamapsychiatry.2017.0303 28384733 PMC5693377

[37] Samuels K, McClellan MB, Patel K, Darling M. Transforming Rural Health Care: High-Quality, Sustainable Access to Specialty Care [Internet]. Brookings. 2001 [cited 2020 Jan 21]. Available from: https://www.brookings.edu/opinions/transforming-rural-health-care-high-quality-sustainable-access-to-specialty-care/

[38] Schottenfeld JR, Waldman SA, Gluck AR, Tobin DG. Pain and Addiction in Specialty and Primary Care: The Bookends of a Crisis. J Law Med Ethics J Am Soc Law Med Ethics. 2018;46(2):220–37. 10.1177/1073110518782923 30146986

[39] Finley EP, Mader M, Haro EK, Noël PH, Bernardy N, Rosen CS, et al Use of Guideline-Recommended Treatments for PTSD Among Community-Based Providers in Texas and Vermont: Implications for the Veterans Choice Program. J Behav Health Serv Res. 2019 4 1;46(2):217–33. 10.1007/s11414-018-9613-z 29748747

[40] MacDowell M, Glasser M, Fitts M, Nielsen K, Hunsaker M. A national view of rural health workforce issues in the USA. Rural Remote Health. 2010;10(3):1531 20658893 PMC3760483

[41] Wiznia DH, Zaki T, Maisano J, Kim C-Y, Halaszynski TM, Leslie MP. Influence of Medical Insurance Under the Affordable Care Act on Access to Pain Management of the Trauma Patient. Reg Anesth Pain Med. 2017;42(1):39–44. 10.1097/AAP.0000000000000502 27776094 PMC5173388

[42] Tick H, Nielsen A, Pelletier KR, Bonakdar R, Simmons S, Glick R, et al Evidence-Based Nonpharmacologic Strategies for Comprehensive Pain Care: The Consortium Pain Task Force White Paper. EXPLORE. 2018 5 1;14(3):177–211. 10.1016/j.explore.2018.02.001 29735382

[43] Schatman ME. The Role of the Health Insurance Industry in Perpetuating Suboptimal Pain Management. Pain Med. 2011 3 1;12(3):415–26. 10.1111/j.1526-4637.2011.01061.x 21332933

[44] Becker WC, Dorflinger L, Edmond SN, Islam L, Heapy AA, Fraenkel L. Barriers and facilitators to use of non-pharmacological treatments in chronic pain. BMC Fam Pract [Internet]. 2017 3 20 [cited 2020 Jan 21];18. Available from: https://www.ncbi.nlm.nih.gov/pmc/articles/PMC5359906/10.1186/s12875-017-0608-2 PMC5359906 28320337

[45] Heyward J, Jones CM, Compton WM, Lin DH, Losby JL, Murimi IB, et al Coverage of Nonpharmacologic Treatments for Low Back Pain Among US Public and Private Insurers. JAMA Netw Open. 2018 10 5;1(6):e183044–e183044. 10.1001/jamanetworkopen.2018.3044 30646222 PMC6324451

[46] Pacini D, Murana G, Leone A, Di Marco L, Pantaleo A. The Value and Limitations of Guidelines, Expert Consensus, and Registries on the Management of Patients with Thoracic Aortic Disease. Korean J Thorac Cardiovasc Surg. 2016 12 5;49(6):413–20. 10.5090/kjtcs.2016.49.6.413 27965917 PMC5147465

[47] Donohoe H, Stellefson M, Tennant B. Advantages and Limitations of the e-Delphi Technique: Implications for Health Education Researchers. Am J Health Educ. 2012 1;43(1):38–46.

[48] Annual Surveillance Report of Drug-Related Risks and Outcomes—United States, 2017. Surveillance Special Report 1. Centers for Disease Control and Prevention. U.S. Department of Health and Human Services.; 2017 Aug.

[49] Gomes T, Tadrous M, Mamdani MM, Paterson JM, Juurlink DN. The Burden of Opioid-Related Mortality in the United States. JAMA Netw Open. 2018 6 1;1(2):e180217 10.1001/jamanetworkopen.2018.0217 30646062 PMC6324425

[50] Lin LA, Bohnert ASB, Kerns RD, Clay MA, Ganoczy D, Ilgen MA. Impact of the Opioid Safety Initiative on opioid-related prescribing in veterans. Pain. 2017;158(5):833–9. 10.1097/j.pain.0000000000000837 28240996

[51] United States, Congress. VA Prescription Data Accountability Act 2017 [Internet]. Stat., 115–86 2017 p. 1276. Available from: https://www.congress.gov/bill/115th-congress/house-bill/1545

[52] Saloner B, McGinty EE, Beletsky L, Bluthenthal R, Beyrer C, Botticelli M, et al A Public Health Strategy for the Opioid Crisis. Public Health Rep Wash DC 1974. 2018 12;133(1_suppl):24S–34S. 10.1177/0033354918793627 PMC6243441 30426871

